# Treatment of Typhoid Fever

**Published:** 1891-10

**Authors:** W. B. Cox

**Affiliations:** Sandsford, Chester co., S. C.


### TREATMENT OF TYPHOID FEVER.

BY W. B. COX, M. D., SANDSFORD, S. C.

Typhoid fever is regarded as an acute self-limited disease with a local manifestation. Hence the sooner we recognise it the better. My rule is when called to see a patient with fever, and who gives a history of general *malaise* for a few days previous to taking his bed, is to "clean out" his alimentary canal with some mild cathartic or gentle laxative. If his temperature be under 103, I defer all antipyretic treatment. In the meantime, however, I endeavor to keep his secretions active. My reason for deferring all antipyretic treatment, is that I may observe his temperature independently of antipyretic influence, thereby enabling me to sooner reach a conclusion as to the proper classification of his fever. Classified as typhoid, the following is my treatment:

At the outset I stated that typhoid fever is self-limited—hence no specific treatment. Indeed, the physician who intelligently does the least is the most successful.

First and foremost, maintain the strength of your patient and keep his temperature within safe limits. As to the rest of the treatment, it is altogether symptomatic.

As a diet for patients with typhoid fever, I regard sweet milk, *par excellence.* In itself, it is capable of maintaining the strength of a patient almost indefinitely. I always insist that he drink, at least, from one to one and one-half quarts daily, to be taken at intervals of about four hours. In the latter stages of the disease, I order whiskey to be taken at short intervals—enough to increase the strength, but not the frequency of the pulse. Frequently patients tell me they can't drink sweet milk, in fact never could. To that I always reply, "Well, just regard it as so much medicine and take it anyhow." In addition to sweet milk, I also allow chicken broths, gruels, poached eggs, egg-noggs, buttermilk, etc.; but, from the commencement of the fever to established convalescence, I confine my patient to a strictly liquid diet.

Antipyretic treatment. I endeavor to keep the temperature under 103 by sponging with cold water and by administering antifebrine and quinine, either singly or conjointly. When my patient has a nurse with sufficient intelligence to use a thermometer, I prescribe four or five grains of antifebrine to be given every hour, until the temperature falls to the desired point, say 101 or 102; but, if my patient has not a nurse with sufficient intelligence to use a thermometer, I prescribe four or five grains of antifebrine with one or two grains of sulphate of quinine to be given every four hours, to be increased or diminished according to indications. From theoretical considerations, salol is the ideal antipyretic for typhoid fever, since it is antiseptic as well as antipyretic; but, as yet, I have no experience with it in this disease. Of all the antipyretics for typhoid fever, I believe that cold water is the safest and the best; but the difficulties and inconveniences attending its application in the rural districts, precludes its general use—certain high authorities to the contrary.

Keep the emunctories active. Quinine, antifebrine, and sponging with cold water generally maintains functional activity of the skin. The bowels are kept open with oil and turpentine, and by enemas of warm water. There must be one good evacuation daily, but never more than three. Should diarrhœa supervene, I like nothing better than the old time enema of "starch water and laudanum." To relieve tympany and the pain incident thereto, turpentine stupes, of course, is the treatment: but I confess that I have often seen them do very little or no good. For that condition, I prefer small doses of tincture of nux vomica, frequently repeated. More or less nervous depression is the natural accompaniment of typhoid fever. Being a general nerve stimulant, nux vomica, by maintaining functional activity of the nerve centers, does good in many ways. For instance, it not only relieves but prevents gastric irritability, a frequent concomitant of typhoid fever; and it excites and maintains peristalsis, and in that way not only relieves but prevents tympany. Other examples in its favor could easily be mentioned. Just here, however, I will mention one theoretical objection to its use. A local intestinal inflammation is one of the characteristics of typhoid fever, and in all inflammatory conditions rest, surgical rest, is one of the first principles of treatment. Hence anything that would increase peristalsis would produce a condition of *unrest*, a condition at once inconsistent with surgical *rest*. So the administration of nux vomica, theoretically considered, is simply choosing the lesser of two evils.

Renal and respiratory activity is maintained indirectly by the above treatment. In alluding to renal activity, I must not forget the time-honored turpentine. If anything approaches a specific for typhoid fever, I think turpentine does. It not only does good by keeping up renal activity, but is an excitant of the secretions generally. Being an efficient antiseptic and hœmostatic, its good effects in typhoid fever are easily appreciated.

Now, as to the nurse and sick-room. I endeavor to have two nurses, one to serve during the day and the other during the night. Tell them they must carry out your directions to the letter. If they have no experience in nursing, and, especially in nursing your patients, it is better to reduce your directions to writing, at least for the first few days. If possible, no article of food or drink must be kept in the sick room. Place the bed in a position where the patient can get as much fresh air as possible, but at the same time not exposed to a draught. Keep clean, dry clothes on patient, and have bedding changed as frequently as practicable. If the patient complains of headache have cold cloths applied to the head.

A few words to the physician. Give as few opiates as possible. In all fevers there is more or less inactivity of the secretions. Opiates check the secretions. Hence by their administration you produce the very condition that you should endeavor to prevent. When administering antifebrine, be extremely cautious as to the use of opiates. Alarming symptoms have been produced by their conjoined administration. The reason I employ antifebrine as an antipyretic, is its cheapness—a very important item, where a physician furnishes his own medicine, and to young physicians in particular. See your patients as often as possible.

Now, in conclusion let me say that I make no claims to originality in outlining this course of treatment, in regard to which I could say a great deal more, did space permit. It is a course of treatment with which I have been uniformly successful. Being a young physician, and appreciating the obstacles and difficulties encountered by the same, I write this, trusting that something said may be of some service to lonely young physicians struggling to reach the pinnacle of professional fame.

W. B. COX, M. D.,
Sandsford, Chester co., S. C.

